# Association between volume of lung damage and endoplasmic reticulum stress expression among severe COVID-19 ICU patients

**DOI:** 10.3389/fmed.2024.1368031

**Published:** 2024-06-11

**Authors:** Domitille Renard, Mikael Verdalle-Cazes, Perrine Leprêtre, Jérémy Bellien, Valery Brunel, Sylvanie Renet, Fabienne Tamion, Emmanuel Besnier, Thomas Clavier

**Affiliations:** ^1^Department of Anesthesiology and Critical Care, CHU Rouen, Rouen, France; ^2^Department of Radiology, CHU Rouen, Rouen, France; ^3^INSERM EnVI UMR 1096, University of Rouen Normandie, Rouen, France; ^4^Department of Pharmacology, CHU Rouen, Rouen, France; ^5^Department of General Biochemistry, CHU Rouen, Rouen, France; ^6^Medical Intensive Care Unit, CHU Rouen, Rouen, France

**Keywords:** COVID-19, endoplasmic reticulum stress, inflammation, interleukin-6, acute respiratory distress syndrome, organ failure, humans, intensive care unit

## Abstract

**Introduction:**

Links have been established between SARS-CoV-2 and endoplasmic reticulum stress (ERS). However, the relationships between inflammation, ERS, and the volume of organ damage are not well known in humans. The aim of this study was to explore whether ERS explains lung damage volume (LDV) among COVID-19 patients admitted to the intensive care unit (ICU).

**Materials and methods:**

We conducted a single-center retrospective study (ancillary analysis of a prospective cohort) including severe COVID-19 ICU patients who had a chest computed tomography (CT) scan 24 h before/after admission to assess LDV. We performed two multivariate linear regression models to identify factors associated with plasma levels of 78 kDa-Glucose-Regulated Protein (GRP78; ERS marker) and Interleukin-6 (IL-6; inflammation marker) at admission.

**Results:**

Among 63 patients analyzed, GRP78 plasma level was associated with LDV in both multivariate models (β = 22.23 [4.08;40.38]; *p* = 0.0179, β = 20.47 [0.74;40.20]; *p* = 0.0423) but not with organ failure (Sequential Organ Failure Assessment (SOFA) score) at admission (r = 0.03 [−0.22;0.28]; *p* = 0.2559). GRP78 plasma level was lower among ICU survivors (1539.4 [1139.2;1941.1] vs. 1714.2 [1555.2;2579.1] pg./mL. respectively; *p* = 0.0297). IL-6 plasma level was associated with SOFA score at admission in both multivariate models (β = 136.60 [65.50;207.70]; *p* = 0.0003, β = 193.70 [116.60;270.90]; *p* < 0.0001) but not with LDV (*r* = 0.13 [−0.14;0.39]; *p* = 0.3219). IL-6 plasma level was not different between ICU survivors and non-survivors (12.2 [6.0;43.7] vs. 30.4 [12.9;69.7] pg./mL. respectively; *p* = 0.1857). There was no correlation between GRP78 and IL-6 plasma levels (*r* = 0.13 [−0.13;0.37]; *p* = 0.3106).

**Conclusion:**

Among severe COVID-19 patients, ERS was associated with LDV but not with systemic inflammation, while systemic inflammation was associated with organ failure but not with LDV.

## 1 Introduction

The emergence of severe acute respiratory syndrome coronavirus 2 (SARS-CoV-2) disease (COVID-19) at the end of 2019 has affected 689 million people worldwide and has been responsible for 6.9 million deaths (data from 28 March 2023) (1). This virus mainly induces pulmonary damages that can lead to acute respiratory distress syndrome (ARDS) through cell apoptosis and inflammatory storm (2, 3). This inflammatory stress is linked to pro-inflammatory cytokines, notably interleukin-6 (IL-6), which is associated with organ failure among COVID-19 patients (4, 5). The volume of these pulmonary lesions induced by SARS-CoV-2 is assessed by chest computed tomography (CT), which is now the reference exam for COVID-19 patients. The extent of SARS-CoV-2 lesions on CT is known to be predictive of severity, regardless of analytical methods used (scoring, ventilation imaging, etc.) (6, 7).

Endoplasmic reticulum (ER) stress (ERS) and its adaptive response, the unfolded protein response (UPR), represent an archetypal example of adaptive stress response. The ER plays a crucial role in protein folding, notably regulated by specific proteins known as chaperones, such as the 78 kDa Glucose-Regulated Protein (GRP78) which stimulates the correct folding of polypeptide to functional protein complexes (8). Multiple disturbances observed during infection or trauma can result in a dysfunction of the ER, leading to the accumulation of unfolded proteins within the lumen of the ER, known as ERS (9, 10). One of the roles of the UPR is to lead the synthesis of new chaperones to allow protein folding (e.g., GRP78, the final effector of UPR). However, if the ERS is severe and prolonged, UPR can lead to cell death by apoptosis (11). Several studies have shown that GRP78 and UPR mediators are enhanced in ARDS patients, suggesting that ERS may be a central component of lung inflammatory diseases. In comparison to healthy individuals, COVID-19 patients had higher plasma levels of GRP78, higher expression of GRP78 within pneumocytes and alveolar macrophages (12–14). It was also mentioned an increase in GRP78 expression on the surface of leukocytes in severe COVID-19 patients, a correlation between circulating GRP78 and COVID-19 severity among COVID-19 patients and amelioration of the lung hyperinflammatory response by the modulation of ERS in a mouse COVID-19 ARDS model (15). Some data suggested that GRP78 acts as a pro-viral protein during SARS-CoV-2 infection (16). However, we recently found no association between GRP78 plasma level and clinical worsening from a “severe” toward a “critical” condition in ICU patients (17).

ERS is classically linked to systemic inflammation and organ failure (18, 19). However, a recent study questioned the causal relationship between inflammation, organ injury and ERS during sepsis. Thus, in a model of surgical peritoneal sepsis [cecal ligation-puncture (CLP)], peritoneal infection predominantly triggered inflammatory responses, while damages induced by surgical stress were predominant triggers of the ERS/UPR response (20). These data, showing that inflammation was little associated with tissue trauma (but driven by infection) while ERS/UPR were essentially driven by tissue trauma more than by infection or systemic inflammation, raise the question of the relationship between ERS, inflammation and volume of organ damage (cellular necrosis/tissue degradation).

To our knowledge, while several works reported links between organ failure, inflammation and ERS among humans, there is no study which directly assessed the link between the volume of organ damage and ERS expression. Therefore, the aim of this study was to investigate whether circulating GRP78 explains the volume of pulmonary damage among patients with severe COVID-19 admitted in ICU.

## 2 Materials and methods

### 2.1 Study design

We conducted a single-center retrospective study using a previously prospectively collected data set. The present study is an ancillary analysis of the COVID-THELIUM cohort, that was described in a previous publication (21). This cohort concerned severe COVID-19 patients hospitalized in three intensive care units (ICU) of a tertiary care hospital between May and November 2020. This cohort was approved by an ethics committee (Comité de Protection des Personnes Est-III, approval number 2020-A00885-34). According to the French law, verbal approval was required from the patient or their relatives (22). The study was conducted in accordance with the principles of the Declaration of Helsinki related to human research. The elaboration of the manuscript was in accordance with the STROBE statement.

To put things briefly, patients were eligible for inclusion if they presented a “severe” condition related to a documented infection by SARS-CoV-2 (determined by polymerase chain reaction assay), defined as the need for ICU admission because of acute respiratory failure. Non-inclusion criteria were pregnancy, a documented bacterial co-infection, known limitations in life support because of patient choice or important comorbidities and an expected death within 24 h. For the present study, we also excluded patients who did not receive a lung scan within 24 h before or after ICU admission and patients already on mechanical ventilation at ICU admission.

Clinical data were collected at admission and during ICU stay, including conventional characteristics, Sequential Organ Failure Assessment (SOFA) score, conventional biological characteristics (blood count, creatinine, C-reactive protein (CRP), aspartate aminotransferase (AST), lactatemia, high-sensitivity troponin T). Patients were followed for up to 28 days or until death.

### 2.2 Endpoints

Our primary endpoint was to investigate whether circulating GRP78 plasma level explain lung damage volume (expressed in percentage of total lung volume) evaluated by chest CT.

Our secondary endpoints were to investigate an association between:

- GRP78 plasma level and organ failure and inflammation;
- IL-6 plasma level and organ failure, tissue damage and inflammation;
- GRP78 and IL-6 plasma levels and length of ICU/hospital stay and mortality.

### 2.3 CT scan

Chest CT scans were re-analyzed in DICOM format by Thoracic VCAR software (GE Healthcare, Chicago, Illinois, United States) for every patient (23). This new analysis was performed by a single radiologist, blinded to the patients’ condition and biological results. Soft tissue reconstruction was used before this analysis. This software allows an automatic segmentation of the two lungs. On this segmentation, a thresholding by binarization was applied, assuming the darkest voxels correspond to air (healthy alveoli, bronchial and bronchiolar lumens) and the lightest voxels to the damaged territory (ground glass hyperdensity, alveolar condensations). The threshold used for binarization was −522UH, which seems to have the best diagnostic value for COVID-19 (24). The software displayed the volume and percentage of involvement for each lung. The volume of damage was then expressed as a percentage of total lung volume for each patient.

### 2.4 Blood sample and assays

Whole blood was collected on ethylenediaminetetraacetic acid (EDTA) tubes within the first 24 h after ICU admission, using a venous or arterial catheter. Tubes were immediately centrifugated for 15 min (2000 G, 4°C) and plasma was frozen at −20°C for a maximum of 2 weeks before being frozen at −80°C until final assays.

Assays were performed using an Enzyme-Linked Immunosorbent Assay for GRP78 (Enzo Life Sciences, Villeurbanne, France), and using an Electro-Chemiluminescence Immunoassay for IL-6 (Roche Diagnostics, Meylan, France).

### 2.5 Data and statistical analysis

Because of the non-normal distribution of data, as observed using a Shapiro–Wilk test, results are expressed as medians with interquartile ranges (IQR) for quantitative data and as absolute numbers and percentages (n, %) for qualitative data. Analysis was performed using Spearman correlation test or Mann–Whitney test according to the type of variable. In cases of missing data, no value was imputed.

We conducted univariate analyses to investigate which factors were associated with GRP78 and IL-6 plasma levels in our study. We then established two multivariate linear regression models with GRP78 and IL-6 plasma levels as associated factors with the endpoints studied:

- The first one took into account factors associated with GRP78 or IL-6 plasma levels in univariate analysis. The significance level to include a parameter in the model was 0.1 (*a posteriori* model);
- The second was an *a priori* theoretical model including factors that were clinically relevant or frequently found associated with GRP78 and/or IL-6 plasma levels in medical literature (15, 25–28). We included in this *a priori* theoretical model: demographic characteristics (age, body mass index (BMI)), medical history (hypertension, diabetes), volume of lung damaged by SARS-CoV-2 (% of total lung volume), inflammatory markers used in daily practice (neutrophils, CRP) and quantification of organ failure (SOFA score, lactates).

Statistical significance was assessed using an alpha level of 0.05. The statistical analyses were performed by means of the statistical software GRAPHPAD PRISM (9.5.1).

## 3 Results

### 3.1 Patient’s characteristics

A total of 98 patients were included in the initial COVID-THELIUM cohort, 35 were excluded (timing of chest CT unknown or more than 24 h before/after ICU admission and/or already under mechanical ventilation at admission) leading to 63 patients analyzed. Their main clinical and biological characteristics at ICU admission are shown in Table 1.

**Table 1:** Characteristics of the population analyzed.

	Parameters	All (*n* = 63)
Anthropometric parameters and medical history at ICU admission	Age (years)	66.0 [57.0;74.0]
	Male	46.0 (63.0)
	Body mass index (kg/m^2^)	30.1 [24.9;33.8]
	Hypertension	38.0 (60.0)
	Diabetes	30.0 (48.0)
	Delay between first COVID-19 symptoms and ICU admission (days)	8.0 [5.5;10.0]
Pulmonary parameters and scores at ICU admission	Lung damage volume (%)	33.0 [27.0;41.0]
	Pulmonary embolism	6.0 (11.0)
	PaO_2_/FiO_2_ ratio	135.0 [97.5;168.0]
	SOFA score day 0	33.0 [26.5;40.0]
Clinical evolution	Intubation	22.0 (34.9)
	Prone	14.0 (22.2)
	Catecholamine	16.0 (25.4)
	Length of ICU stay (days)	9.0 [5.5;19.0]
	Length of hospital stay (days)	19.0 [10.5;31.7]
	Death in ICU	7.0 (11.0)
	Death in hospital	7.0 (11.0)
	SOFA score day 7	1.0 [0.0;3.7]
Biological parameters at ICU admission	Leukocytes (G/L)	7.4 [5.8;10.2]
	Polynuclear neutrophils (G/L)	6.5 [4.4;8.9]
	Lymphocytes (G/L)	0.6 [0.5;0.9]
	D-dimer (ng/mL)	1142.5 [796.8;1985.2]
	Lactates (mmol/L)	1.3 [0.9;1.7]
	AST (UI/L)	46.5 [34.2;67.7]
	Prothrombin time (%)	99.0 [88.5;100.0]
	Creatinine (μmol/L)	78.0 [55.5;95.5]
	CRP (mg/L)	114.0 [63.0;187.5]
	Troponin (ng/L)	14.0 [8.5;23.5]
	GRP78 plasma level (pg/mL)	1564.1 [1162.2;1969.4]
	IL-6 plasma level (pg/mL)	12.5 [7.5;46.2]

Data are presented as medians with interquartile range or absolute values and percentages (n, %). ICU, intensive care unit; SOFA score, Sequential Organ Failure Assessment score; AST, Aspartate Aminotransferase; CRP, C-reactive protein; GRP78, 78 kDa Glucose-Regulated Protein; IL-6, Interleukin 6.

### 3.2 Endoplasmic reticulum stress marker

**Figure 1:**
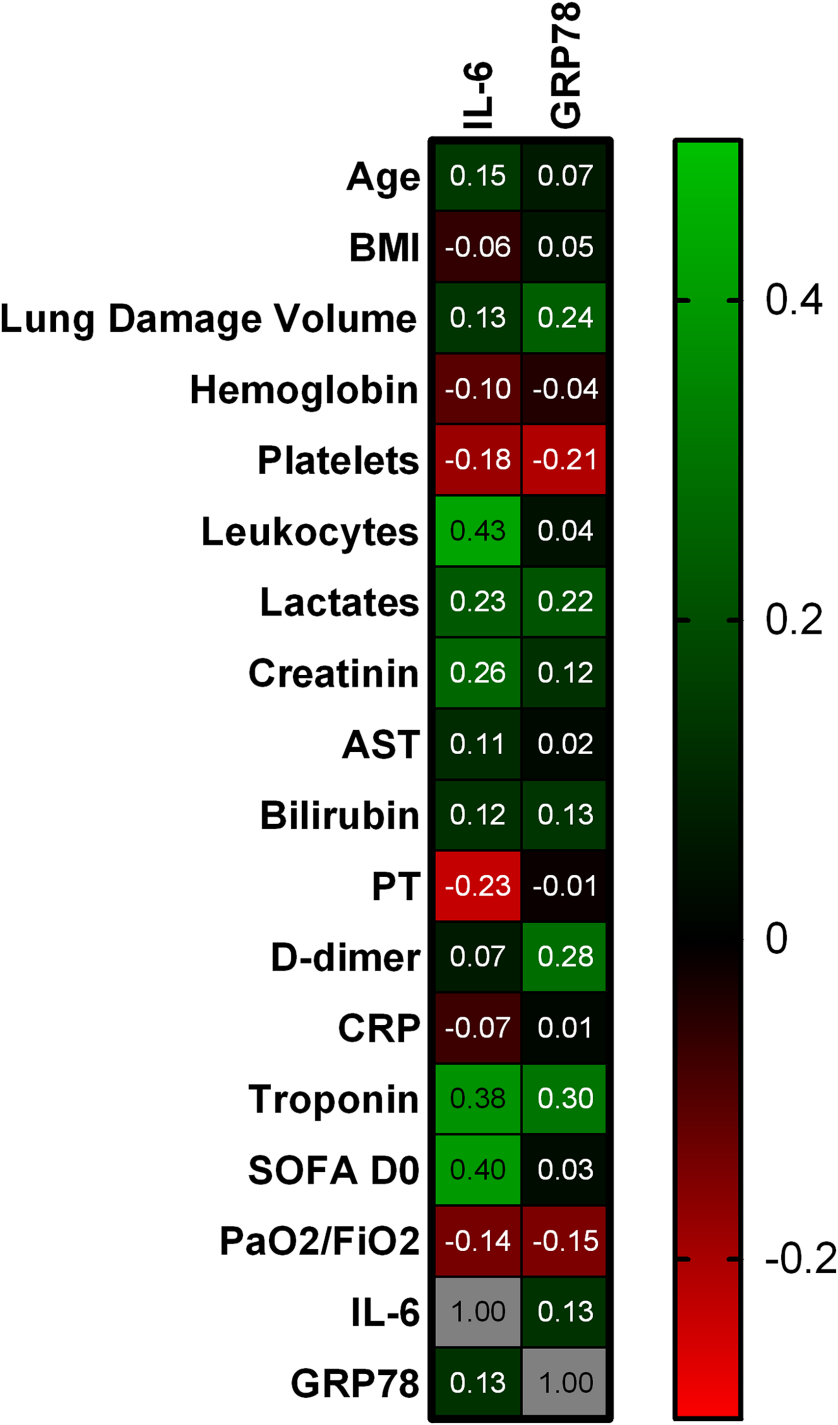
Correlation between characteristics at ICU admission and GRP78/IL-6 plasma levels. This figure presents the Spearman r correlation coefficient between quantitative parameters and GRP78/IL6 plasma levels at admission. Green represents a positive correlation with intensity increasing according to the lightness of the green, black represents no correlation, and red represents a negative correlation, with intensity increasing according to the lightness of the red. BMI, Body Mass Index; AST, Aspartate Aminotransferase; PT, Prothrombin Time; CRP, C-Reactive Protein; SOFA D0 score, Sequential Organ Failure Assessment day 0 score; IL-6, Interleukin-6; GRP78, 78 kDa Glucose-Regulated Protein.

Quantitative parameters associated with GRP78 plasma level in univariate analysis are shown in Figure 1. Detailed results with 95% confidence interval are given in Supplementary material 1. Pulmonary embolism on chest CT was associated with higher GRP78 plasma level (1949.0 [1865.0;2083.0] vs. 1507.6 [1139.2;1877.8] pg./mL; *p* = 0.0298) but we found no association between GRP78 plasma level and gender, and history of diabetes or hypertension.

The multivariate regression model based on univariate analyses results, including platelets, lactates, D-dimer, troponin, pulmonary embolism and lung damage volume, only found an association between lung damage volume percentage and GRP78 plasma level (Table 2). The multivariate regression model based on clinically relevant or frequently described factors also found an association between lung damage volume percentage and GRP78 plasma level (Table 2).

**Table 2:** Linear regression models (factors associated with GRP78 plasma level).

Variable	β coefficient [95% CI]	*p*-value
**“**Model” based on univariate analyses		
Platelets	−1.03 [−2.50;0.43]	0.1618
Lactates	9.25 [−357.70;376.20]	0.9594
D-dimer	0.01 [−0.09;0.13]	0.7810
Troponin	0.47 [−11.87;12.81]	0.9382
Pulmonary embolism	416.6 [−333.7;1167.0]	0.2667
Lung damage volume	22.23 [4.08;40.8]	0.0179
“Model” based on clinically relevant and known factors associated with GRP78/IL-6 plasma levels		
Age	15.10 [−2.86;33.07]	0.0974
BMI	−9.64 [−47.89;28.61]	0.6144
Hypertension	−129.70 [−582.70;323.30]	0.5673
Diabetes	20.81 [−402.30;443.90]	0.9216
SOFA score day 0	19.33 [−78.99;117.70]	0.6943
Lung damage volume	20.47 [0.74;40.20]	0.0423
Polynuclear neutrophils	4.55 [−45.22;54.32]	0.8548
CRP	−1.68 [−4.14;0.77]	0.1743
Lactates	159.20 [−210.20; 528.70]	0.3902

GRP78, 78 kDa Glucose-Regulated Protein; BMI, Body Mass Index; SOFA score, Sequential Organ Failure Assessment score; CRP, C-Reactive Protein.

### 3.3 Inflammatory marker

Quantitative parameters associated with IL-6 plasma level in univariate analysis are shown in Figure 1. Detailed results with 95% confidence interval are given in Supplementary material 2. We found no association between IL-6 plasma level and pulmonary embolism, gender, history of diabetes or hypertension.

The multivariate regression model based on univariate analyses, including leukocytes, lactates, creatinine, prothrombin time, troponin and SOFA score at day 0, only found an association between SOFA score at day 0 and IL-6 plasma level (Table 3). The multivariate regression model based on clinically relevant or frequently described factors also found an association between SOFA score at day 0 and IL-6 plasma level (Table 3).

**Table 3:** Linear regression models (factors associated with IL-6 plasma level).

Variable	β coefficient [95% CI]	*p*-value
“Model” based on univariate analyses		
Leukocytes	20.41 [−13.13;53.96]	0.2270
Lactates	101.5 [−156.0;359.1]	0.4318
Creatinine	−2.27 [−3.08;2.54]	0.8472
Prothrombin time	12.24 [−2.92;27.40]	0.1109
Troponine	−2.76 [−9.05;3.52]	0.3810
SOFA score day 0	193.7 [116.6;270.9]	< 0.0001
“Model” based on clinically relevant and known factors associated with IL-6/GRP78 plasma levels		
Age	−2.37 [−15.36;10.62]	0.7154
BMI	19.84 [−7.82;47.50]	0.1557
Hypertension	−171.20 [−498.80;156.30]	0.2984
Diabetes	203.90 [−102.10;509.80]	0.1865
SOFA score day 0	136.60 [65.50;207.70]	0.0003
Lung damage volume	−6.11 [−20.38;8.15]	0.3930
Polynuclear neutrophils	26.63 [−9.36;62.61]	0.1433
CRP	0.03 [−1.74;1.81]	0.9681
Lactates	124.80 [−142.30;392.00]	0.3520

IL-6, Interleukin-6; SOFA score, Sequential Organ Failure Assessment score; BMI, Body Mass Index; CRP, C-Reactive Protein.

### 3.4 GRP78/IL-6 plasma levels and clinical evolution

There was no correlation between IL-6 plasma level (reflecting systemic inflammation) and GRP78 plasma level (reflecting intensity of ERS expression; *r* = 0.13 [−0.13;0.37]; *p* = 0.3106).

We found an association between IL-6 plasma level and length of ICU stay (*r* = 0.55 [0.34;0.70] *p* < 0.0001), length of hospital stay (*r* = 0.36 [0.11;0.56]; *p* = 0.0045), and SOFA score at day 7 (*r* = 0.60 [0.40;0.74]; *p* < 0.0001), but no association between these parameters and GRP78 plasma level.

Regarding the association between GRP78/IL-6 plasma levels and qualitative variables, we found an association between GRP78 plasma level and death, and between IL-6 plasma level and the need for intubation, prone positioning, and catecholamine administration (Table 4).

**Table 4:** Association between GRP78 and IL-6 plasma levels with morbidity and mortality criteria.

		GRP78 (pg/mL)		IL-6 (pg/mL)	
	N (%)	Median [IQR]	*p*-value	Median [IQR]	*p*-value
Intubation	Yes: 22.0 (34.9)	1589.3 [1326.8;2164.6]	0.5788	42.9 [19.9;73.0]	0.0001
	No: 41.0 (65.1)	1539.4 [1139.2;1941.1]		10.8 [4.1;22.5]	
Prone	Yes: 14.0 (22.2)	1639.6 [1478.6;2036.5]	0.3945	48.4 [32.5;680.0]	0.0002
	No: 49.0 (77.8)	1559.8 [1141.6;1950.7]		11.4 [4.7;30.8]	
Catecholamine	Yes: 16.0 (25.4)	1592.5 [1436.0;1946.0]	0.4344	44.5 [23.1;301.6]	0.0007
	No: 47.0 (74.6)	1541.4 [1136.8;1969.4]		11.4 [4.7;31.6]	
Death (ICU or hospital)	Yes: 7.0 (11.1)	1714.2 [1555.2;2579.1]	0.0297	30.4 [12.9;69.7]	0.1857
	No: 56.0 (88.9)	1539.4 [1139.2;1941.1]		12.2 [6.0;43.7]	

GRP78, 78 kDa Glucose-Regulated Protein; IL-6, Interleukin-6; ICU, intensive care unit.

## 4 Discussion

Our results suggest that ERS and its response, UPR, could be independently associated with the volume of lung damage in severe COVID-19 patients and explain it. We did not find any association between GRP78 plasma level and inflammation or systemic organ failure. IL-6 plasma level seemed independently associated with systemic organ failure but not with tissue injury. Finally, IL-6 plasma level was more correlated with short-term severity while GRP78 plasma level was associated with short-term mortality. We developed two models of multivariate analysis for GRP78 and IL-6 plasma levels. One with strictly associated parameters in univariate analyses, the second one taking into account relevant factors found in the literature. The aim was to take into account as many confounding factors as possible, including those not found in our study and to strengthen the value of our findings with a “double methodology.” We distinguish between lung lesion volume, which represents a volume of tissue damage without presuming its functionality, and organ failure, represented by the SOFA score, which objectively reflects organ dysfunction.

Our objective was to study a “clinical model” of infectious injury associated with systemic inflammation in humans, involving a single organ whose volume of injured tissue could be easily quantified *in vivo* with a reliable and validated method. Studying a population of patients with severe COVID-19 allowed us to have a population with a pathology meeting these criteria. Indeed, SARS-CoV-2 has an essentially lung tropism responsible for an isolated respiratory failure and whose organ damages are easy to quantify *in vivo* by validated CT methods, with nevertheless an important systemic inflammation (2, 5, 29). We used a dedicated software used by a single radiologist to estimate lung damage through binarization. The analysis was then automatic, allowing us to avoid the human factor: the radiologist did not interpret the CT scans but only used the software. We arbitrarily chose a 24-h window between CT and the measurement of GRP78 plasma level. Our objective being to study the link between pulmonary damage and ERS and the kinetics of circulating GRP78 being a few hours, this 24-h window with a new systematic analysis by a blinded operator of previously performed chest CT seemed relevant (30).

Upregulation of circulating GRP78 is specific to ERS response and is considered to be a sensitive marker of ERS intensity (8). Furthermore, elevated blood and lung GRP78 levels had already been found among COVID-19 patients (13, 14). In our cohort, the lowest GRP78 plasma level was 406.3 pg./mL with a median at 1564.1 [1162.2;1969.4] pg./mL, which is well above the thresholds found in studies investigating the diagnostic values of GRP78 plasma level. Recently a threshold of GRP78 plasma level at 157.3 pg./mL was thus suggested for sepsis diagnosis (sensitivity 75.0%; specificity 73.1%) (31). A recent work studying inflammation and ERS among COVID-19 patients suggested that any SARS-CoV-2 positive patient who showed a GRP78 plasma level superior to 300 pg./mL in serum at the beginning of the infection had a 100% probability of developing pneumonia (15). The same study suggested that the plasma level of GRP78 among COVID-19 patients was associated with organ failure and severity of infection while we did not find an association between organ failure and GRP78 plasma level (15). It appears in agreement with our previous results in this cohort, which found no association between GRP78 plasma level at admission and clinical worsening in ICU (17). However, our patients had much higher plasma levels of GRP78 than the vast majority of this previous cohort, suggesting that this association could be influenced by the infection severity. As the GRP78 plasma level at admission was higher in deceased patients’ plasma than in survivors’, our results are however compatible with the link between ERS expression and short-term prognosis among COVID-19 patients. High plasma levels of IL-6 are known to be associated with adverse clinical outcomes, including ICU admission, ARDS, mechanical ventilation and death among COVID-19 patients (4, 32). We found an association between IL-6 plasma level and organ failure as previously described, showing that our population is similar to previous cohorts of COVID-19 patients admitted in ICU.

In our cohort, GRP78 plasma level was associated with lung damage volume but not with inflammation or organ failure, while IL-6 plasma level was associated with organ failure but not with lung damage volume or ERS. To respond to ERS, cells activate an adaptive pathway, the UPR, to synthesize chaperones (including GRP78) and restore normal ER function. While a strong UPR appears necessary in the acute stress phase, excessively prolonged ERS responses promote cell death as a result of an imbalance in favor of pro-apoptotic pathways rather than anti-apoptotic pathways (11). The fact that ERS and then UPR may promote apoptosis could be an explanation for the link between high GRP78 plasma levels and macroscopic volume of damaged organ. It is known that organ and/or tissue damage without infection can lead to ERS expression in humans and we previously described that among humans with surgical stress, there was no correlation between GRP78 and IL-6 or CRP levels (30, 33, 34). However, it is also often presumed in animal models that inflammation triggers ERS response (and vice versa). Our findings are in concordance with the recent animal study by Müllebner et al. showing that peritoneal infection induced by CLP predominantly triggered inflammatory response, while damages caused by surgery were predominant triggers of the ERS/UPR response (20). They hypothesized that ERS/UPR and inflammatory response *in vivo* during sepsis may be triggered by different mechanisms and that secondary tissue injury influences the severity of ERS more than the intensity of local and systemic inflammation. The association found between GRP78 plasma level and lung damage volume in our cohort combined with a non-correlation between ERS and systemic inflammation (IL-6) strengthen their hypothesis and describe for the first time this potential link among infected humans. This hypothesis is also comforted by recent studies in mouse models showing that important mechanical volume induced lung injury was the predominant trigger of ERS, preceding and contributing to lung inflammation (35, 36). As it is known that ERS expression after tissue injury last longer than systemic inflammation in humans, and given its association with damaged lung volume, it would be interesting to study the link between ERS expression and long-term prognosis among COVID-19 patients (fibrosis, bronchiectasis, chronic dyspnea,…) (33). Given these recent data, it also probably appears relevant in animal models of septic shock exploring ERS expression, to take into account the existence or not of tissue damage in the model to better interpret the intensity of ERS and UPR.

Regarding inflammation in our patients, we must emphasize that our patients exhibited inflammation with a median CRP of 114 mg/L and median neutrophil count of 6.5 G/L. There is no defined norm to describe a state of hyperinflammation; our patients seem to display an inflammatory state that could be considered moderate. We must also note that although interleukin-6 is a well-recognized and studied marker of inflammation, it also has functions outside of inflammation, such as in hematopoiesis, liver and neuronal regeneration, embryonal development, and fertility (4, 5, 37).

Finally, this dichotomy between ERS on one hand and tissue lesion volume (without presuming organ dysfunction) and on the other hand inflammation and organ failure allows for a different perspective. While inflammation is commonly associated with severity, as we find in the short term in our study, this severity and inflammation do not seem to be correlated with or responsible for the volume of tissue injury. Thus, we can infer that the response to ERS reflects direct viral tissue damage rather than excessive inflammation. ERS intensity could therefore be used as a proxy and marker of tissue lesions during pathology, allowing for the monitoring of lesion evolution.

Despite interesting results, our study has several limitations. Firstly, it was a monocenter retrospective study, including a limited number of patients, even though we had few missing data given the prospective design of the initial cohort. Secondly, some factors that we did not take into account could have impacted the plasma levels of GRP78 and/or IL-6. For example, it has been established in animal models that caloric restriction influences GRP78 production, which is a frequent situation within 24 h of arrival in ICU (38). Thirdly, the maximum lung parenchymal damage is known to occur 11 days after the onset of symptoms in COVID-19 patients, which is also the timing corresponding to their clinical worsening (8–12 days) (7, 26). The median time from symptoms to ICU admission in our population was 8.0 [5.7;10.0] days. We chose to include only patients with a chest CT performed in a 24-h window before/after ICU admission to account for GRP78 plasma level sample timing, but we probably missed the maximum of lung damage and could perhaps have shown a different association by changing this 24-h window. Fourthly, unlike many previous analyses, we did not find an association between IL-6 and CRP plasma levels. Nevertheless, IL-6 plasma level remained correlated with leukocytes, as did CRP. We thus hypothesize that the de-correlation between IL-6 plasma level and CRP could be due to a lack of statistical power. In addition, it appears that there may be some de-correlation between CRP and IL-6 plasma level in the most severe patients, CRP increasing little with a spike at 72 h in contrast to IL-6 plasma level, which increases a lot in a few hours (39). We took this point into account by including CRP in our *a priori* linear regression model. Fifthly, given our methodology, our results do not presume any causality link between ERS and lung tissue damage in humans. While some animal studies described that ERS expression is induced by tissue damage rather than by inflammation or infection, we cannot here confirm this hypothesis among infected humans. Sixth, our samples were taken at only one-time point (ICU admission) and we did not explore the kinetics or ERS/UPR and its links to the evolution of lung lesions during ICU stay. Further studies are needed to understand the kinetics of GRP78 plasma level during infections and sepsis among humans, and its association with organ damage and organ failure. Finally, our models include a large number of variables. However, we also performed the analyses with fewer variables but still keeping lung lesion volume and GRP78 levels, and the associations were still present, allowing us to think that our models are not affected by overfitting.

## 5 Conclusion

Our results suggest that, among severe COVID-19 patients, ERS is associated with the volume of lung damage but not with systemic inflammation, while systemic inflammation is associated with organ failure but not with lung damage. Given the ERS association with organ damage, it would be interesting to study its relationship with long-term functional outcome among COVID-19 patients.

## Data availability statement

The data analyzed in this study is subject to the following licenses/restrictions: Data would be available depending of the reason. Requests to access these datasets should be directed to DR, dmt.renard@gmail.com.

## Ethics statement

The studies involving humans were approved by Comité de Protection des Personnes Est-III, approval number 2020-A00885-34. The studies were conducted in accordance with the local legislation and institutional requirements. Written informed consent for participation was not required from the participants or the participants’ legal guardians/next of kin because according to the French law, verbal approval was required from the patient or their relatives. A written informed consent was not required for this study. Written informed consent was not obtained from the individual(s) for the publication of any potentially identifiable images or data included in this article because according to the French law, verbal approval was required from the patient or their relatives, a written informed consent for publication was not required.

## Author contributions

DR: Writing – original draft, Writing – review & editing. MV-C: Writing – review & editing. PL: Writing – review & editing. JB: Writing – review & editing. VB: Writing – review & editing. SR: Writing – review & editing. FT: Writing – review & editing. EB: Writing – review & editing. TC: Writing – review & editing.
