# Supplementary material for: Association between volume of lung damage and endoplasmic reticulum stress expression among severe COVID-19 ICU patients
Source: Front Med (Lausanne). 2024 Jun 11;11:1368031. doi: 10.3389/fmed.2024.1368031 (PMC11200928; doi:10.3389/fmed.2024.1368031)
Supplement: Supplementary file 1 [file Table_1.DOCX]

**Supplementary Material 1. Correlation between characteristics at ICU admission and GRP78 plasma level.**

|  | **Correlation with GRP78 plasma level:**  **coefficient [confidence interval]** | **p-value** |
| --- | --- | --- |
| Age | 0.06 [-0.19;0.31] | 0.6114 |
| BMI | 0.05 [-0.20;0.30] | 0.6733 |
| Hemoglobin | -0.04 [-0.29;0.21] | 0.7456 |
| Platelets | -0.20 [0.44;0.04] | 0.1002 |
| Leukocytes | 0.04 [-0.21;0.29] | 0.7312 |
| Polynuclear neutrophils | 0.08 [-0.17;0.33] | 0.5148 |
| Lactates | 0.22 [-0.04;0.45] | 0.0918 |
| Creatinine | 0.12 [-0.13;0.36] | 0.3358 |
| AST | 0.02 [-0.23;0.28] | 0.8592 |
| Prothrombin time | -0.01 [-0.27;0.24] | 0.9240 |
| D-Dimer | 0.28 [0.02;0.51] | 0.0317 |
| Fibrinogen | -0.06 [-0.31;0.20] | 0.6483 |
| CRP | 0.01 [-0.24;0.27] | 0.9117 |
| Troponin | 0.30 [0.03;0.53] | 0.0257 |
| SOFA score day 0 | 0.03 [-0.22;0.28] | 0.8089 |
| PaO2/FiO2 ratio | -0.15 [-0.39;0.11] | 0.2559 |
| Lung damage volume | 0.24 [-0.03;0.48] | 0.0723 |

GRP78 : 78kDa Glucose-Regulated Protein, BMI : Body Mass Index, AST : Aspartate Aminotransferase, CRP : C-Reactive Protein, SOFA score : Sequential Organ Failure Assessment score.
