# Supplementary material for: Association between volume of lung damage and endoplasmic reticulum stress expression among severe COVID-19 ICU patients
Source: Front Med (Lausanne). 2024 Jun 11;11:1368031. doi: 10.3389/fmed.2024.1368031 (PMC11200928; doi:10.3389/fmed.2024.1368031)
Supplement: Supplementary file 2 [file Table_2.DOCX]

**Supplementary Material 2. Correlation between characteristics at ICU admission and IL-6 plasma level.**

|  | **Correlation with IL-6 plasma level :**  **coefficient [confidence interval]** | **p-value** |
| --- | --- | --- |
| Age | 0.15 [-0.11;0.39] | 0.2488 |
| BMI | -0.05 [-0.31;0.20] | 0.6423 |
| Hemoglobin | -0.10 [-0.34;0.15] | 0.4179 |
| Platelets | -0.18 [-0.42;0.07] | 0.1527 |
| Leukocytes | 0.42 [0.19;0.61] | 0.0005 |
| Polynuclear neutrophils | 0.46 [0.23;0.64] | 0.0002 |
| Lactates | 0.23 [-0.03;0.46] | 0.0717 |
| Creatinine | 0.26 [0.01;0.48] | 0.0370 |
| AST | 0.11 [-0.15;0.36] | 0.3972 |
| Prothrombin time | -0.23 [-0.46;0.02] | 0.0682 |
| D-Dimer | 0.06 [-0.20;0.32] | 0.6245 |
| Fibrinogen | 0.10 [-0.16;0.35] | 0.4295 |
| CRP | -0.07 [-0.32;0.19] | 0.5779 |
| Troponin | 0.38 [0.12;0.59] | 0.0043 |
| SOFA score day 0 | 0.40 [0.16;0.59] | 0.0012 |
| PaO2/FiO2 ratio | -0.14 [-0.38;0.12] | 0.2856 |
| Lung damage volume | 0.13 [-0.14;0.39] | 0.3219 |

IL-6 : Interleukin-6, BMI : Body Mass Index, AST : Aspartate Aminotransferase, CRP : C-Reactive Protein, SOFA score : Sequential Organ Failure Assessment score.
